# Enhancement of immunogenicity of SARS-CoV-2 spike protein expressed in *Escherichia coli* by fusion of the CRM197 functional domain

**DOI:** 10.3389/fmicb.2025.1650239

**Published:** 2025-08-12

**Authors:** Xibing Yu, Yinmeng Yang, Miao Zhang, Qiantong Shen, Yun Zhu, Tong An, Siqi Li, Kexin Zhang, Shuaiyao Lu, Shaohong Lu, Fangcheng Zhuang, Meng Gao

**Affiliations:** ^1^Zhejiang Key Laboratory of High-level Biosafety and Biomedical Transformation, Hangzhou Medical College, Hangzhou, China; ^2^Engineering Research Center of Novel Vaccine of Zhejiang Province, Hangzhou Medical College, Hangzhou, China; ^3^Institute of Medical Biology, Chinese Academy of Medical Sciences and Peking Union Medical College, Kunming, China

**Keywords:** CRM197, SARS-CoV-2, S protein, RBD, vaccines, adjuvant

## Abstract

As an immunogenic non-toxic mutant of diphtheria toxin (DT), cross-reacting material 197 (CRM197) exhibits a significant immunogenicity-enhancing effect on various pathogenic vaccines. However, its application in vaccines against highly pathogenic pathogens remains underexplored. In this study, we incorporated CRM197 into a severe acute respiratory syndrome coronavirus 2 (SARS-CoV-2) vaccine, aiming to identify a molecular adjuvant capable of inducing broad-spectrum neutralizing antibodies and to develop a subunit vaccine with stronger cross-reactivity, improved safety, and greater accessibility. Our findings revealed that compared to non-fused SARS-CoV-2 spike (S) protein receptor-binding domain (RBD) (designated sRBD), the fusion of CRM197 with the SARS-CoV-2 S protein RBD (termed CRM-RBD) elicited stronger humoral immunity, Th1-biased cellular immune responses, and reduced immune evasion against SARS-CoV-2 variants in mice. Furthermore, mice immunized with CRM-RBD exhibited significantly lower mortality and reduced pulmonary pathology upon viral challenge. These results demonstrate that CRM197 substantially enhances the immunogenicity of SARS-CoV-2 vaccines, positioning it as an ideal candidate protein for developing SARS-CoV-2 vaccines with broader cross-reactivity.

## Introduction

As a non-toxic mutant of diphtheria toxin (DT), cross-reacting material 197 (CRM197) is derived from the catalytic C domain of DT harboring a mutation at position 52 (Gly to Ala) (Malito et al., 2012). This protein exhibits broad utility, including its ability to synergistically induce both humoral and cellular immune responses *in vivo* (Jiang et al., 2023). Additionally, CRM197 can be fused with target proteins to enhance their immunogenicity (Liu L. et al., 2022). As a molecular adjuvant, CRM197 has been widely incorporated into vaccine development against various viral pathogens. For instance, in human cytomegalovirus (HCMV) vaccine research, a novel peptide vaccine was designed by conjugating peptide epitopes to the CRM197 carrier protein (Jiang et al., 2023). Similarly, fusion of CRM197 with influenza A virus antigens has been shown to improve vaccine immunogenicity (Xu et al., 2021). However, studies on the application of CRM197 in vaccines against highly pathogenic pathogens remain limited.

Severe acute respiratory syndrome coronavirus 2 (SARS-CoV-2), the highly pathogenic agent responsible for coronavirus disease 2019 (COVID-19), has posed a significant challenge to global public health (Wu et al., 2020). Consequently, the development of effective vaccines remains a critical strategy for COVID-19 prevention. Currently, most SARS-CoV-2 vaccines under development target the spike (S) protein as the primary antigen (Lee and Kim, 2022). The S protein consists of S1 and S2 subunits, which mediate viral attachment to host cell surfaces, leading to membrane fusion and subsequent viral entry. Notably, the receptor-binding domain (RBD) within the S1 subunit recognizes and binds to the host receptor angiotensin-converting enzyme 2 (ACE2) (Wang et al., 2020).

Unfortunately, during the ongoing pandemic, SARS-CoV-2 has continually evolved, giving rise to multiple variants of concern (VOCs), including the B.1.1.7 lineage (Alpha variant), B.1.617.2 lineage (Delta variant), and BA.1/B.1.1.529 lineage (Omicron variant). These variants exhibit an alarming capacity to evade vaccine-induced neutralizing antibodies (Garcia-Beltran et al., 2022), raising serious concerns about the potential emergence of a variant capable of entirely escaping existing vaccines and therapeutic antibodies. To address this challenge, it is imperative to develop next-generation SARS-CoV-2 vaccines with broader cross-reactivity, improved safety, and enhanced accessibility to confer more comprehensive immunity. Therefore, in addition to eliciting protective antibodies, vaccine strategies should aim to enhance immune responses, such as T cell-mediated immunity or heterologous effects driven by innate immune mechanisms (Sauer and Harris, 2020). For subunit vaccines, the incorporation of appropriate adjuvants represents a critical approach to augmenting protective efficacy (Dai et al., 2020).

Researchers have identified potential cross-reactivity between CRM197 and SARS-CoV-2 proteins, which may confer protection against SARS-CoV-2 infection and mortality (Root-Bernstein, 2020). Additionally, some studies have explored fusing CRM197 with SARS-CoV-2 S protein structural domains to enhance neutralizing antibody (NAb) levels in mice. However, a notable limitation is that the neutralization antibody titers of candidate vaccines were assessed solely using a vesicular stomatitis virus (VSV) pseudovirus-based neutralization assay (employing ACE2-expressing BHK21 cells), without validation through authentic SARS-CoV-2 virus neutralization tests (Liu L. et al., 2022).

Therefore, we hypothesize that CRM197 may function as an intramolecular adjuvant to enhance the immunogenicity of the SARS-CoV-2 RBD. In this study, we constructed a fusion protein (designated CRM-RBD) expressed in *Escherichia coli*, comprising CRM197 (Met1-Arg187) and the SARS-CoV-2 RBD (Asn331-Glu583). Subsequently, we conducted a comparative analysis of the biochemical properties, antigenicity, immunogenicity, and protective efficacy between CRM-RBD and the non-fused sRBD (Asn331-Glu583). Based on the research findings, it has been conclusively determined that incorporating CRM197 represents an ideal direction for developing novel SARS-CoV-2 vaccines.

## Materials and methods

### Strains, plasmid, and cell line

The SARS-CoV-2 prototype strain (GD108: GDPCC-nCOV27) and Delta variant (B.1.167.2) used in this study were obtained from the National Kunming High-level Biosafety Primate Research Center, Institute of Medical Biology, Chinese Academy of Medical Sciences. The pET28a (+) plasmid and BL21 (DE3) *Escherichia coli (E. coli)* strains were purchased from Invitrogen (Carlsbad, CA, United States). Vero E6 cells were purchased from American Type Culture Collection (Manassas, VA, United States) and maintained in minimum essential medium (MEM) supplemented with 10% fetal bovine serum (Gibco, Thermo Scientific, United States), in a humidified atmosphere of 5% CO_2_ at 37°C.

### Construction of the fusion protein plasmid

**Figure 1:**
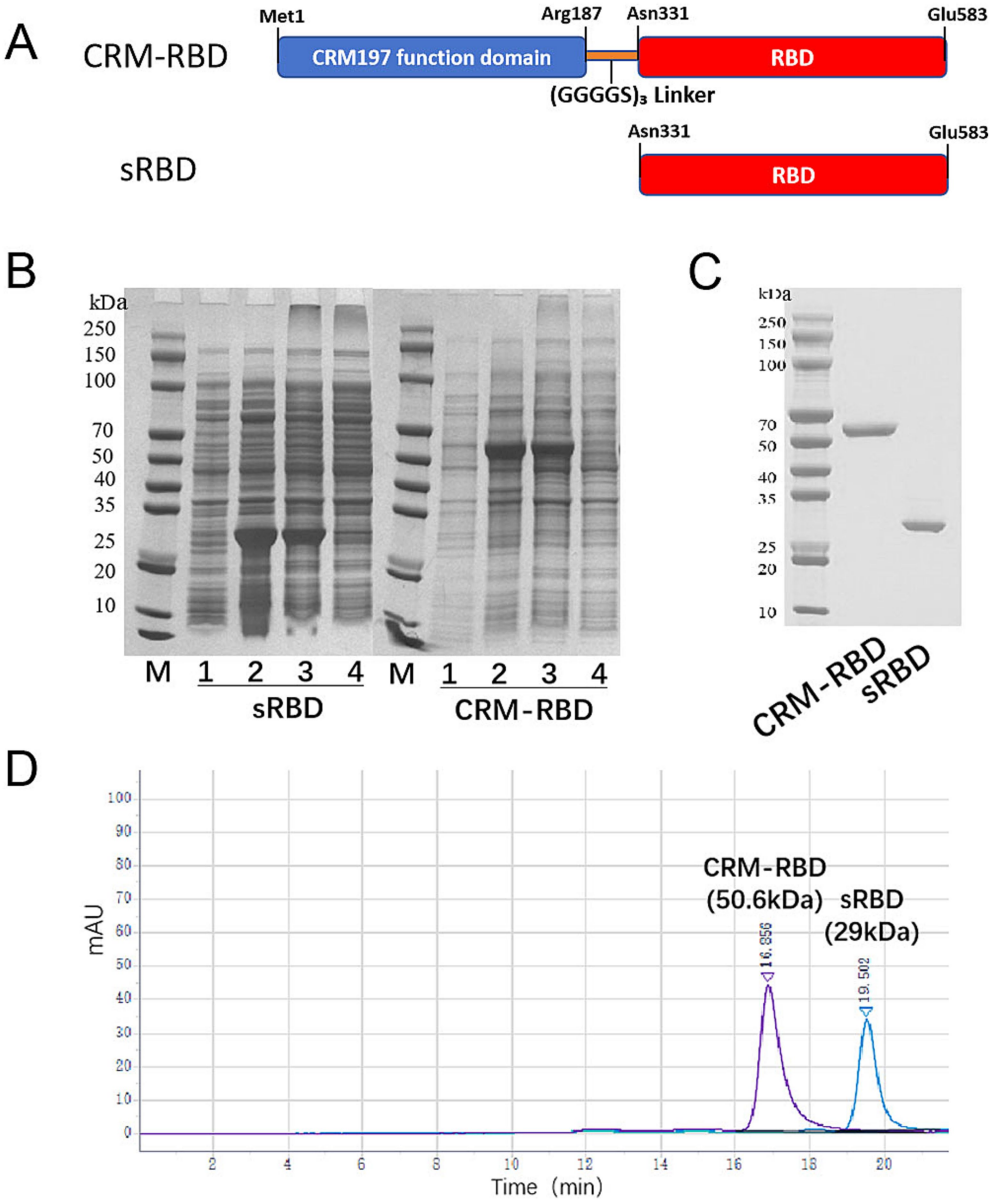
Generation of the sRBD and CRM-RBD proteins. **(A)** Schematic diagram of sRBD and CRM-RBD sequences. The sRBD spans residues Asn331-Glu583 in the S1 domain of SARS-CoV-2. The CRM-RBD contains the CRM197 functional domain (Met1-Arg187) fused to the N-terminus of sRBD with a (GGGGS)_3_ linker. The coding sequences of sRBD and CRM-RBD were subcloned into a pET28a vector to construct recombinant plasmids. **(B)** Expression of sRBD and CRM-RBD proteins in *E. coli*. Lanes on the polyacrylamide gel are labeled as M: marker; 1: non-induced bacterial proteins; 2: induced bacterial proteins; 3: inclusion body pellet; 4: centrifuged supernatant. **(C)** SDS-PAGE analysis of purified sRBD and CRM-RBD. **(D)** HP-SEC analysis of purified sRBD and CRM-RBD. All experiments were performed in triplicate.

The recombinant RBD protein (designated sRBD) was derived from residues Asn331-Glu583 of the SARS-CoV-2 S protein (GenBank accession No. YP_009724390.1, Supplementary Table S1). To ensure that the RBD domain in CRM-RBD can fold freely and can expose critical epitopes, this study constructed the fusion protein by linking the sRBD sequence to the CRM197 functional domain (amino acids 1–187, AMV91693.1, Supplementary Table S1) via a (GGGGS)_3_ linker, forming CRM-RBD (Figure 1A). Both sRBD and CRM-RBD genes were codon-optimized using computational algorithms (provided by Tsingke, Beijing, China) that analyzed codon degeneracy and usage frequency, and were subsequently commercially synthesized by the same company. The optimized genes were subsequently cloned into the pET28a(+) plasmid vector between *BamHI* and *EcoRI* (New England Biolabs, Massachusetts, United States) restriction sites.

### Expression, purification, and preparation of the fusion protein

For protein expression, each plasmid was verified by sequencing (Tsingke, China) before being transformed into BL21 (DE3) cells. Isopropyl β-D-1-thiogalactopyranoside (IPTG)-induced (1 mmol/L) protein expression was conducted in a 50 L fermenter at 25°C for 4 h. The bacteria were harvested by centrifugation (6, 000 g, 10 min, 4°C), followed by resuspension in buffer A (20 mmol/L Tris–HCl, 1 mmol/L EDTA, 500 mM NaCl, pH 8.5) and homogenization (600–800 bar, 3 cycles) using a nano homogenizer (AH100D; ATS, BVI, Canada). After centrifugation (15,000 rpm, 15 min, 4°C), the pellets were washed twice with buffer B (20 mmol/L Tris–HCl, 1 mmol/L EDTA, 500 mM NaCl, 0.1% TrintonX-114, pH 8.5) and dissolved in the denaturing buffer (20 mmol/L Tris–HCl, 8 mol/L urea, pH 7.0). The denatured protein was purified using Capto S (Cytiva, United States) ion-exchange chromatography (IEX) and diethylaminoethyl cellulose (DEAE) (Cytiva, United States) IEX, successively. The protein was diluted to 100 μg/mL and then subjected to renaturation using a Tangential Flow Filtration (TFF) system (Pellicon XL PXB010A50; Biomax, Millipore, Burlington, MA, United States). Specifically, a 10 kDa TFF membrane cassette was employed, with the feed pressure maintained at or below 0.2 bar during ultrafiltration dialysis. This process was performed at 4°C. Following refolding, the protein solution was purified by Superdex200pg (Cytiva, United States) size exclusion chromatography (SEC) and subjected to sterile filtration using 0.2 μm filters (Millipore,). After dilution, the proteins were mixed with aluminum adjuvant (Croda, Denmark) for adsorption.

### Biophysical analysis

The purity and molecular weight of proteins were analyzed by sodium dodecyl sulfate-polyacrylamide gel electrophoresis (SDS-PAGE) and high-performance size-exclusion chromatography (HP-SEC). The endogenous fluorescence emission spectra of the purified protein were recorded with excitation at 280 nm by SpectraMax iD3 (Molecular Devices, United States). The particle size distribution of the purified protein was measured by dynamic light scattering (DLS) using a Zetasizer Nano ZS 90 device (Malvern Instruments, Malvern, UK). Protein morphology was observed under H-7650 transmission electron microscope (TEM) (HITACHI, Japan) at magnification of 400,000 × using phosphotungstic acid staining.

### Antigenicity analysis

Human angiotensin-converting enzyme 2 (hACE-2) protein (Genscript, Nanjing, China) was coated onto 96-well plates at 4°C overnight. Then a 2-fold dilution series of the recombinant protein was added to the plate and incubated at 37°C for 30 min, with protein buffer as control. After 5 washes using phosphate buffered saline containing 0.05% Tween-20 (PBS-T), anti-RBD mouse monoclonal antibody (Sino biological, Beijing, China) diluted 1: 1000 was added to the plates and incubated at 37°C for 60 min. Subsequently, horseradish peroxidase (HRP)-conjugated goat anti-mouse IgG (CWBIO, Beijing, China) diluted 1: 5000 was added as the detection antibody. Finally, 100 μL/well 3,3′,5,5′-tetramethylbenzidine (TMB) and 50 μL/mL H_2_SO_4_ was used as substrate and stop solution, respectively. The color change of the wells of the plate was measured at 450 nm by microplate reader (Molecular Devices, United States).

### Culture, concentration, and titration of SARS-CoV-2 virus

SARS-CoV-2 exhibits high adaptability in Vero E6 cells. The virus was inoculated into confluent Vero E6 monolayers. Upon observation of significant cytopathic effect (CPE) under microscopy (72 h post-infection), the viral supernatant was harvested and stored at −80°C. The following day, the frozen supernatant was thawed at 4°C, clarified by centrifugation, and concentrated via ultrafiltration. The concentrate was washed three times with PBS and eluted to a final volume of 200 mL. Viral titers were determined by plaque assay.

### Enzyme-linked immunosorbent assay (ELISA)

The recombinant RBD protein expressed by 293 T cells (Genscript, Nanjing, China) was diluted to 0.1 μg/mL using a carbonate coating buffer (pH 9.6). Then, 100 μL per well was added to a 96-well microplate and coated overnight at 4°C. Then serially diluted mouse sera (ranging from 1: 10 dilution to 1: 20971520) were added to each well, and then HRP-conjugated goat anti-mouse IgG/IgG1/IgG2a/IgG2b (Abcam, Massachusetts, United States) was added. The TMB color reaction was then used to detect the HRP-conjugated antibody as described for the antigenicity analysis. The resulting titers were calculated from the serial dilution result that represented the highest serum dilution exhibiting OD_450_ ≥ 2.1-fold of the background values.

### Surrogate virus neutralization assay

The Surrogate Virus Neutralization Test kit (GenScript, Nanjing, China) was used to detect competitive inhibition (CI) by antibodies in immunized mouse serum against the binding of RBD to the hACE2 receptor. Briefly, a 1: 500 dilution of mouse serum was incubated with HRP-RBD at room temperature for 30 min, then 50 μL of the serum/RBD mixture was added to each well of hACE2-precoated plates, with serum dilution buffer as the negative control. Each sample was tested in duplicate. After incubation and washing, the OD_450_ values were measured using a microplate reader. The inhibition rate was calculated by the following: (1-sample OD_450_/negative OD_450_) × 100%.

### Live SARS-CoV-2 neutralization assay

Serum samples were collected from mice. Following established protocols (Yang et al., 2020), serum neutralization titers against live SARS-CoV-2 were determined using a CPE-based assay. The serum NAb titer was calculated by assessing CPE 72 h after infecting Vero E6 cells in 96-well plates with 100 TCID_50_ of SARS-CoV-2 at various serum dilution ratios.

### Enzyme-linked immunosorbent spot

The mouse interferon (IFN)-γ enzyme-linked immunosorbent spot (ELISPOT) assay (Dakewe Biotech, Shenzhen, China) was performed according to the manufacturer’s instructions. Briefly, spleen lymphocytes (3 × 10^5^ per well, in duplicate) from immunized mice were plated into an ELISPOT plate and stimulated with an overlapping RBD peptide library (GenScript, Nanjing, China) or phytohemagglutinin (PHA, positive control) in duplicate. After incubation for 20 h in a cell incubator (37°C, 5% CO_2_), the cells were lysed by ice-cold distilled water, and the biotin-conjugated antibody was added to the plate. After incubation for 2 h at room temperature (RT), the spots were developed using streptavidin-HRP and TMB substrate solution. Finally, the spots were counted using an ELISPOT reader (Mabtech, IRIS FluoroSpot, Sweden).

### Mouse vaccinations and sample collection

**Figure 2:**
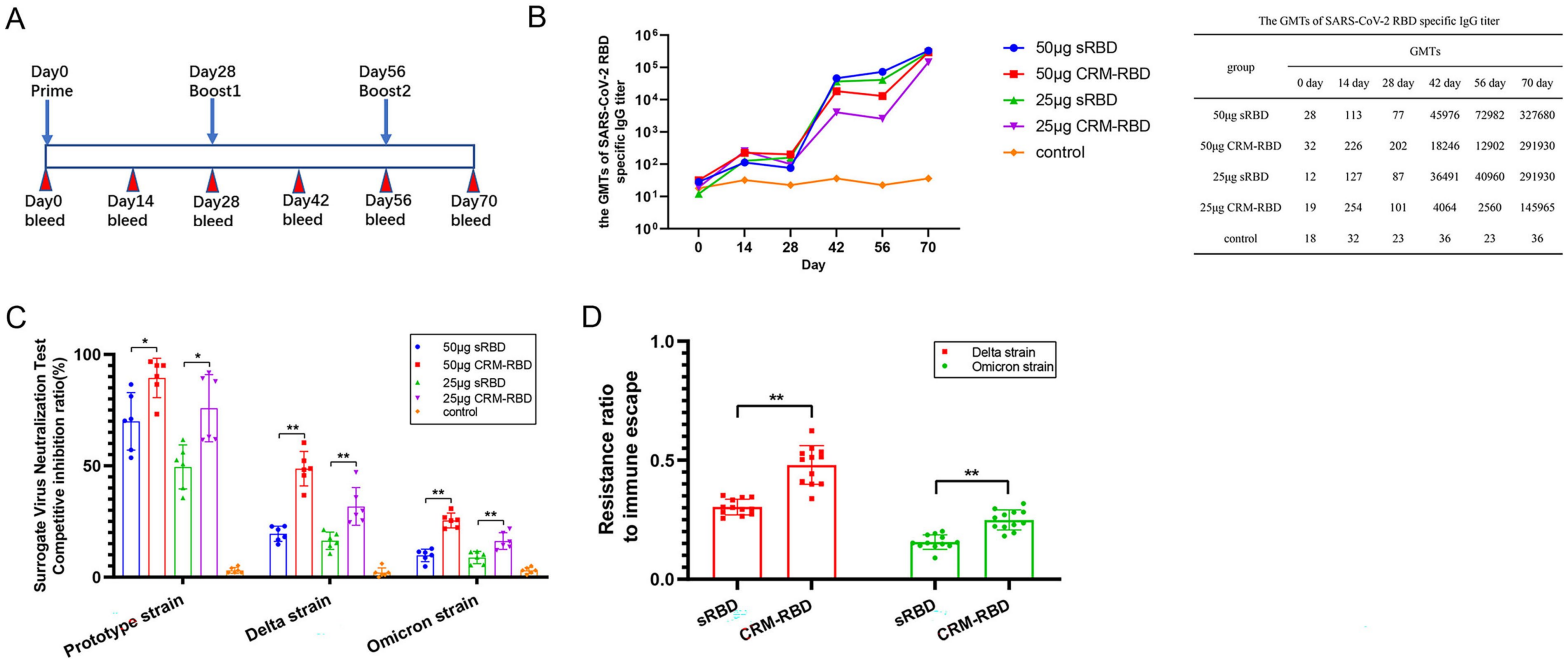
Antibody responses induced by sRBD and CRM-RBD in a mouse immunization study. BALB/c mice were randomly divided into 5 groups (*n* = 6 per group) to receive sRBD or CRM-RBD at 50 μg/dose or 25 μg/dose (with alum adjuvant for protein adsorption), or placebo (alum adjuvant diluted in 0.9% NaCl). Mice received three intraperitoneal doses at 28-day intervals. **(A)** Schematic diagram of the vaccination schedule. **(B)** Serum SARS-CoV-2 RBD-specific antibodies were measured by ELISA every 14 days until day 70. **(C)** The CI rate evaluated by surrogate virus neutralization test kits. Responses were measured against SARS-CoV-2 RBD of prototype, Delta, and Omicron variants. **(D)** The immune escape rate of immunized serum. The ratio of Delta and Omicron variant to prototype CI rate was calculated as the resistance ratio to immune escape. Each experiment was performed in triplicate. *p < 0.05, **p < 0.01, ***p < 0.001, ns (not significant).

Aluminum hydroxide gel manufactured under good manufacturing practice (GMP) conditions was used as an adjuvant (Croda, UK) resulting in alum-precipitated protein. Thirty 4-6-week-old specific-pathogen-free (SPF) BALB/c mice with equal numbers of males and females were then randomly divided into 5 groups. Two groups received 50 μg/dose or 25 μg/dose sRBD in 0.9% NaCl, two groups received 50 μg/dose or 25 μg/dose of CRM-RBD in 0.9% NaCl, and one group was the adjuvant control group that received 0.9% NaCl diluted alum adjuvant. Each group contained six mice. All mice were immunized intraperitoneally (i.p.) with three injections of equal doses at 28-day intervals. During this period (Figure 2A), serum was collected through the eye socket of the mice at 14-day intervals. At the end of the study, which continued until the 70th day, the mice were sacrificed by inhalation of ether and then the neck was broken to collect blood samples and spleen. The specific operation is as follows: Place the dry cotton ball into a small beaker, pour an appropriate amount of ether to completely saturate it, and immediately tighten the cover of the ether container. Place the beaker containing the saturated cotton ball into the anesthesia box, and tightly close the box lid. When the mouse is resting quietly in the cage, grasp the center of its tail with the right hand to lift it up, open the anesthesia box with the left hand, quickly place the mouse into the anesthesia box, and immediately tightly close the box lid. Observe closely, remove it when the mouse spontaneously falls down and its breathing changes from fast to slow, and begin checking its corneal reflex and pain reflex. The disappearance of these reflexes indicates sufficient anesthesia, after which dislocation death is performed. All animals were kept in a sterile environment, and the management and use of animals were approved by the Ethics Review Committee of Hangzhou Medical College. All applicable agency and government regulations regarding the ethical use of animals were followed.

### Mice challenge experiment and related indicator collection

K18-ACE2 transgenic mice (2–4 months old) were provided by GemPharmatech Co., Ltd. (Laboratory Animal Production License: SCXK (Su) 2018-0008). The mice were randomly divided into three immunization groups, and the immunization procedure was performed by the research team following the protocol described in Figure 2A. For the prototype strain challenge experiment, the groups were as follows: CRM-RBD group (50 μg, *n* = 10), CRM-RBD group (25 μg, *n* = 10) and control group (*n* = 10). For the Delta variant challenge experiment, the groups were as follows: CRM-RBD group (50 μg, *n* = 10), CRM-RBD group (25 μg, *n* = 10) and control group (*n* = 20, with 1 death prior to the experiment, resulting in *n* = 19).

**Figure 3:**
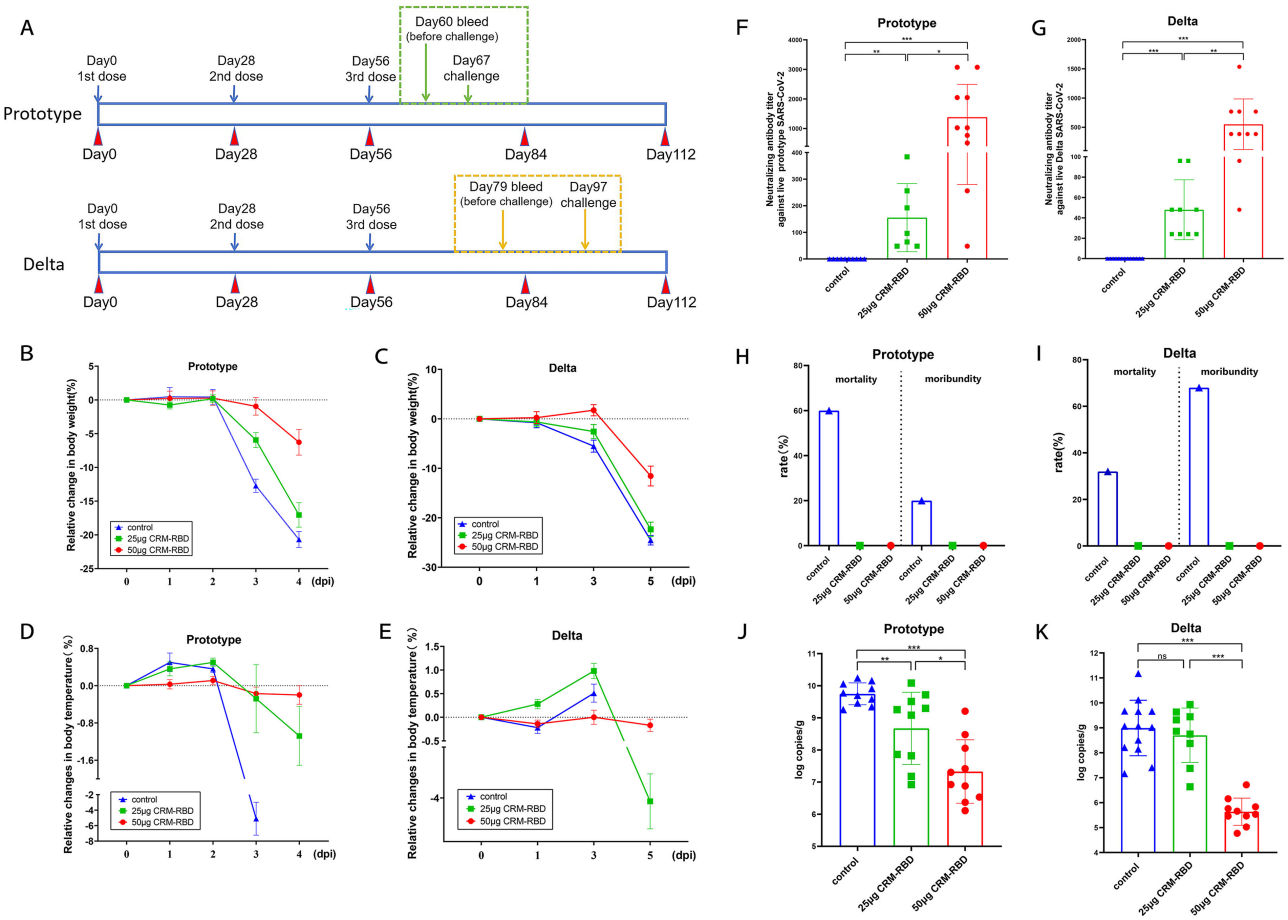
Mouse challenge experiment and indicator detection. Two-to four-month-old K18-ACE2 transgenic mice were randomly divided into three immunization groups: 50 μg CRM-RBD group, 25 μg CRM-RBD group, and control group. Following immunization, challenge experiments were conducted using either the prototype strain or Delta variant of SARS-CoV-2. **(A)** Prototype strain challenge: 10 mice per group. Pre-challenge blood samples were collected on day 60 post-immunization, and challenge was performed on day 67. Delta strain challenge: 10 mice per immunized group, 20 control mice (1 died before the experiment, *n* = 19). Pre-challenge blood samples were collected on day 79, and challenge was performed on day 97. For both strains, clinical parameters (mental status, body weight, and temperature) were monitored daily post-challenge. NAb titers and lung viral loads were assessed on the final day of challenge. **(B,C)** Relative body weight changes after challenge with the prototype **(B)** or Delta **(C)** strain. **(D,E)** Relative temperature changes after challenge with the prototype **(D)** or Delta **(E)** strain. **(F)** NAb titers on day 4 post-challenge with the prototype strain. Control (*n* = 10), 25 μg CRM-RBD (*n* = 7), 50 μg CRM-RBD (*n* = 10). **(G)** NAb titers on day 5 post-challenge with the Delta strain. Control (*n* = 13), 25 μg CRM-RBD (*n* = 9), 50 μg CRM-RBD (*n* = 10). **(H,I)** Mortality and moribund rates after challenge with the prototype **(H)** or Delta **(I)** strain. **(J)** Lung viral load after prototype strain challenge. Control (*n* = 10), 25 μg CRM-RBD (*n* = 10), 50 μg CRM-RBD (*n* = 10). **(K)** Lung viral load after Delta strain challenge. Control (*n* = 13), 25 μg CRM-RBD (*n* = 9), 50 μg CRM-RBD (*n* = 10). All experiments were performed in triplicate. Statistical significance: *p < 0.05, **p < 0.01, ***p < 0.001, ns (not significant).

Blood collection prior to challenge was performed following immunization, followed by challenge experiments. For the Prototype strain: Blood collection (pre-challenge) was performed on day 60 post-immunization. Challenge was conducted on day 67 post-immunization (Figure 3A). Each mouse was challenged via intranasal inoculation with 1 × 10^4^ PFU. For the Delta variant: Blood collection (pre-challenge) was performed on day 79 post-immunization. Challenge was conducted on day 97 post-immunization (Figure 3A). Each mouse was challenged via intranasal inoculation with 1 × 10^4^ TCID_50_.

Following viral challenge, daily observations of murine mental status were conducted alongside body weight and temperature measurements (performed under anesthesia). On the final day of challenge (day 4 post-challenge for the Prototype strain and day 5 post-challenge for the Delta variant), NAb titers were measured across groups. Gross pathological changes in lungs were examined post-dissection. The left lung lobe was fixed for sectioning and hematoxylin and eosin (HE) staining to evaluate histopathological alterations, with a focus on pulmonary tissue pathology. The right lung lobe was harvested for viral load quantification.

All animal infection experiments, including viral challenge, sample collection, and subsequent analyses, were conducted at the National Kunming High-Level Biosafety Non-Human Primate Experimental Center of the Institute of Medical Biology, Chinese Academy of Medical Sciences. Three days prior to viral challenge, all immunized mice were transferred to the ABSL-3 small animal facility for acclimatization. All animal experiments in this study were strictly conducted in compliance with biosafety operational protocols and animal ethics requirements, with humane care provided to ensure animal welfare. All experimental procedures were performed under anesthesia to meet both biosafety requirements and ethical guidelines. The detailed anesthesia protocol was as follows: Infected mice were anesthetized via isoflurane inhalation (2% concentration). Using long forceps, mice were transferred into an anesthesia induction chamber. The anesthesia vaporizer dial and gas flow meter were then adjusted to induce a deep surgical plane of anesthesia. After achieving target anesthesia depth, mice were euthanized by cervical dislocation. If mice exhibited signs of arousal during the procedure, they were re-anesthetized in the induction chamber prior to resuming the operation. The animal study protocol was reviewed and approved by the Institutional Animal Care and Use Committee of the Institute of Medical Biology, Chinese Academy of Medical Sciences [Approval No. DWSP202110023 (Prototype strain) and DWSP202202009 (Delta variant)].

### RNA extraction

Right lung lobes from each experimental group were homogenized and pooled to obtain approximately 100 mg of tissue. The RNA was isolated using the RNA extraction kit (Direct-zol RNA Miniprep Kit, Zymo Research, United States) according to the manufacturer’s protocol. The extracted RNA was eluted in 50 μL of nuclease-free water and stored at −80°C for subsequent one-step quantitative reverse transcription PCR (qRT-PCR) analysis of viral load.

### Quantitative reverse transcription PCR

Viral genomic RNA (gRNA) was quantified using real-time qRT-PCR. Primers and probe targeting the N gene were designed based on sequences recommended by the WHO and the Chinese CDC. The primer sequences are shown in Supplementary Table S2 (FAM is the fluorescent label and BHQ1 is the quencher). The reaction setup, thermal cycling conditions, and dilution of the N gene standard were performed in accordance with the manufacturer’s instructions (TaqMan® Fast Virus 1-Step Master Mix, Thermo Scientific, United States) and standard operating procedures (SOP).

### Pathological analysis

Following euthanasia, the mice’s left lung lobes were collected and fixed in formalin for HE staining and histological examination. Tissue sections were independently evaluated by two blinded pathologists using light microscopy to assess pulmonary histopathological changes. Pathological reports were generated based on microscopic observations, followed by comprehensive scoring of the entire lung lobe histopathology. Vaccine efficacy against viral challenge was ultimately evaluated by integrating the histopathological profiles and composite pathological scores. The scoring table for pulmonary pathological alterations in mice is provided in Supplementary Table S3.

### Statistical analysis

Data were expressed as mean ± standard deviation (SD). Statistical analysis was performed using GraphPad Prism 8.0 (San Diego, CA, United States). The NAb titer was expressed as geometric mean titers (GMT). The related data were statistically analyzed by Mann–Whitney U test for non-parametric data and the paired t test for parametric data. Statistical significance levels are indicated as ns (not significant), * (*p* < 0.05), ** (*p* < 0.01), and *** (*p* < 0.001).

## Results

### Efficient *in vitro* expression and refolding of sRBD and CRM-RBD

The sRBD and CRM-RBD recombinant proteins were successfully expressed in *E. coli*, and their apparent molecular weights were consistent with the theoretical predictions. After homogenization and centrifugation, the recombinant proteins were confirmed to be localized in the inclusion bodies (Figure 1B). As proteins in inclusion bodies are aggregated, correct folding required the sRBD and CRM-RBD proteins to be denatured and renatured to reconstruct their conformation and antigenicity. The renatured sRBD and CRM-RBD monomer proteins were obtained by SEC, and the purity of proteins was determined by SDS-PAGE (Figure 1C) and HP-SEC (Figure 1D). In conclusion, sRBD and CRM-RBD are capable of correct *in vitro* expression and refolding.

### Refolded sRBD and CRM-RBD exhibit proper conformation and competent hACE2 binding capacity *in vitro*

**Figure 4:**
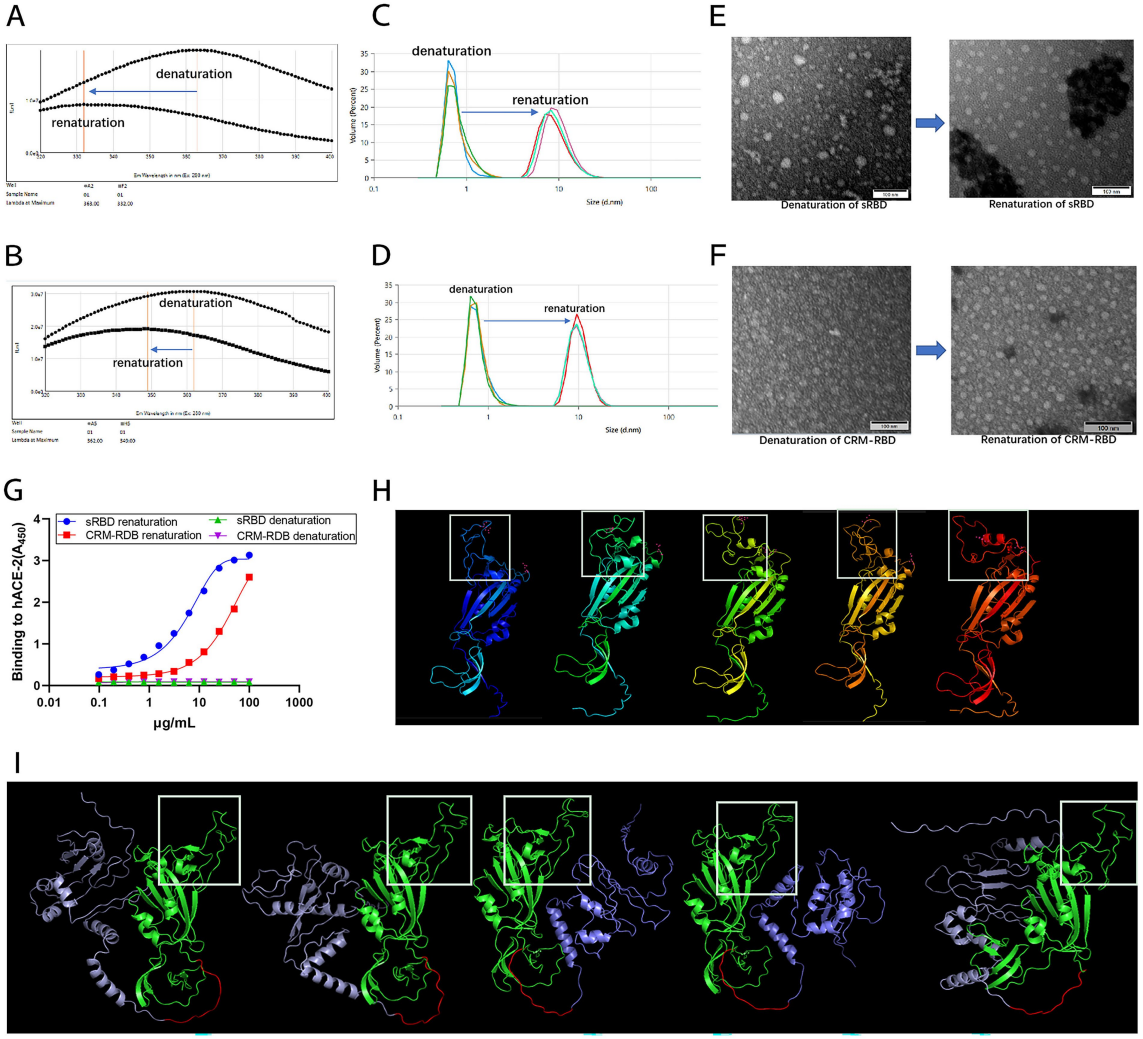
Characterization of the sRBD and CRM-RBD proteins after refolding *in vitro*. To characterize the proteins before and after refolding, we recorded the endogenous fluorescence emission spectra (320–400 nm) with 280 nm excitation, measured the particle size distribution, observed protein morphology using a TEM at 400,000 × magnification, and determined hACE2 and sRBD/CRM-RBD affinity by ELISA. To further investigate the structural properties of sRBD and CRM-RBD, we performed conformational prediction analysis using AlphaFold2. **(A)** The maximum fluorescence emission wavelength (λ_ex280nm_ Max) of sRBD shifted from 363 nm to 332 nm. **(B)** The λ_ex280nm_ Max of CRM-RBD shifted from 362 nm to 349 nm. **(C)** The particle size of sRBD increased from 0.78 ± 0.03 nm to 9.55 ± 0.39 nm. **(D)** The particle size of CRM-RBD increased from 0.76 ± 0.01 nm to 10.29 ± 0.07 nm. **(E,F)** Structural transition of sRBD (E) and CRM-RBD **(F)** from disordered to ordered particles. **(G)** Binding affinity of sRBD and CRM-RBD for hACE2. Each experiment was performed in triplicate. **(H)** Predicted structure of sRBD (white box indicates the RBM region). **(I)** Predicted structure of CRM-RBD (purple: CRM197; red: flexible peptide linker; green: sRBD; white box: RBM region).

Fluorescence emission spectra, DLS, and TEM were used to monitor conformational changes during renaturation (Figure 4). Both sRBD and CRM-RBD exhibited a significant blue shift, with the maximum fluorescence emission wavelength (λ_ex280nm_ Max) ranging from 320 to 400 nm during the transition from denatured to renatured states. For sRBD, the λ_ex280nm_ Max shifted from 363 nm to 332 nm (Figure 4A), while for CRM-RBD, it shifted from 362 nm to 349 nm (Figure 4B). This shift indicated that the aromatic amino acid groups on the side chains of the recombinant proteins (CRM-RBD) were gradually embedded within the molecule (Basak and Chattopadhyay, 2014). DLS analysis revealed that the protein particle diameter of sRBD increased from 0.78 ± 0.03 nm to 9.55 ± 0.39 nm (Figure 4C), and that of CRM-RBD increased from 0.76 ± 0.01 nm to 10.29 ± 0.07 nm (Figure 4D). TEM further confirmed the structural transition from disordered aggregates to well-defined particles (Figures 4E,F). The refolded proteins were subsequently validated for receptor-binding capability using enzyme-linked immunosorbent assay (ELISA) to assess their affinity for hACE2. Both sRBD and CRM-RBD demonstrated dose-dependent binding to hACE2, with half-maximal effective doses (EC_50_) of 0.77 μg/mL for sRBD and 1.45 μg/mL for CRM-RBD (Figure 4G).

To further investigate the structural properties of the sRBD and CRM-RBD recombinant proteins, we performed conformational prediction analysis using AlphaFold2 (Figures 4H,I). The results revealed that the core region of the sRBD protein, particularly the receptor-binding motif (RBM), exhibited high conformational flexibility, with five distinct predicted structural states (Figure 4H). However, upon fusion with CRM197, the RBM region adopted a more stable conformation (Figure 4I), suggesting that the CRM197 domain may contribute to structural stabilization of the sRBD.

These findings demonstrate that prokaryotically expressed CRM-RBD and sRBD maintain proper conformation and demonstrate effective hACE2 binding capacity following *in vitro* refolding. Notably, CRM-RBD appears to adopt a more stable spatial configuration compared to sRBD.

### CRM-RBD-induced neutralizing antibodies reduce immune escape of SARS-CoV-2 variants

To assess the antibody response in mice following immunization with sRBD or CRM-RBD, we measured serum anti-RBD specific antibody titers (GMT) via indirect ELISA using 293 T-expressed RBD. After the primary immunization, GMT levels peaked on day 14 and declined by day 28. Subsequent booster doses (administered on days 28 and 56) elicited robust recall responses, with significant increases in anti-RBD antibody levels observed on days 42 and 70. By day 70, GMT values reached 291, 930 (25 μg sRBD) and 327, 680 (50 μg sRBD), whereas CRM-RBD immunization yielded GMTs of 145, 965 (25 μg) and 291, 930 (50 μg) (Figure 2B).

SARS-CoV-2 primarily enters host cells through RBD interaction with hACE2 receptors. Consequently, the neutralizing capacity of an antibody is determined by its ability to competitively inhibit RBD-hACE2 binding (Sui et al., 2014; Cao et al., 2022). Based on preliminary experiments which demonstrated concordance between NAb titers and serum antibody CI rates across all groups, we ultimately selected the CI assay to ensure measurement stability and sensitivity. Three distinct RBD variants (prototype, Delta, and Omicron strains) were used to evaluate the CI rates of serum antibodies induced by CRM-RBD and sRBD. Compared to control mice, sera from vaccinated mice (50 μg protein dose) demonstrated significant inhibition of RBD-hACE2 binding across all tested variants. Notably, the prototype strain exhibited higher CI rates than mutant variants (Figure 2C). These results indicated that RBD mutations confer partial immune evasion. This study also calculated the CI ratio between variants and prototype strains to determine immune escape rates. For the mutant strains, CRM-RBD demonstrated significantly higher resistance to immune escape compared to sRBD immunization (Figure 2D). Thus, CRM-RBD exhibits superior capability in counteracting immune evasion induced by SARS-CoV-2 variants relative to sRBD.

### Enhanced Th1/Th2 response balance induced by CRM197 RBD in mice

**Figure 5:**
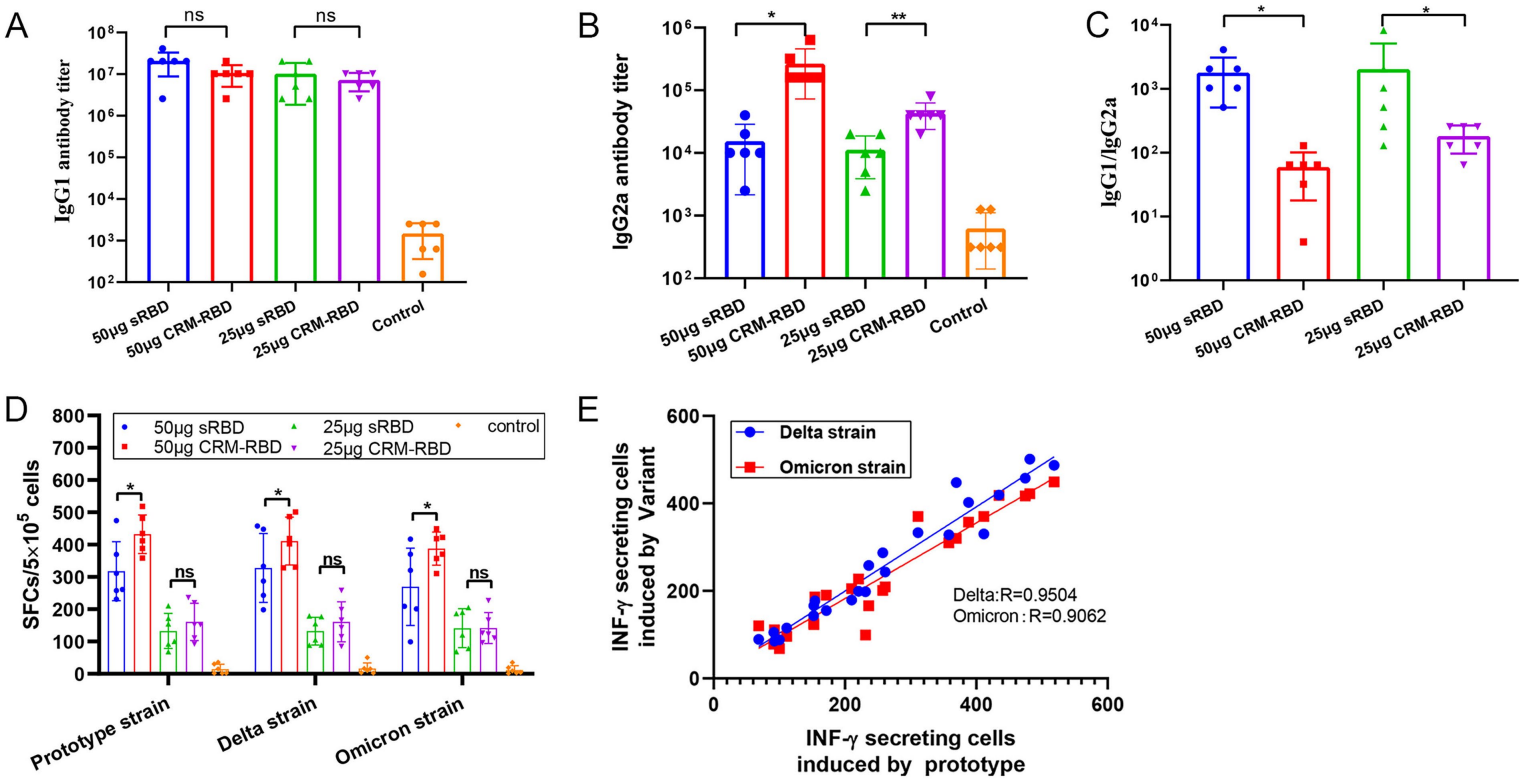
T cell response assay. Serum samples and splenocytes were collected from immunized BALB/c mice 14 days after the third dose. The titers of IgG1 and IgG2a antibody subtypes in serum were quantified by ELISA. Th1-type specific T-cell responses were assessed through IFN-γ ELISpot assay. **(A)** IgG1 titers in serum. **(B)** IgG2a titers in serum. **(C)** IgG1/IgG2a ratio in serum. **(D)** IFN-γ SFCs in splenocytes. **(E)** Correlation between IFN-γ-secreting cells induced by the prototype strain and variants (Delta, Omicron) (R values from linear regression shown). Each experiment was performed in triplicate. *p < 0.05, **p < 0.01, ***p < 0.001, ns (not significant).

To assess Th1/Th2 immune response bias in immunized mice, we measured serum IgG1 and IgG2a antibody titers on day 70. While IgG1 levels showed no significant difference between CRM-RBD and sRBD immunization groups (Figure 5A), CRM-RBD induced markedly higher IgG2a titers compared to sRBD (Figure 5B). Consequently, the IgG1/IgG2a ratio-a key indicator of Th1/Th2 bias-was significantly lower in CRM-RBD-immunized mice than in sRBD controls (Figure 5C). On day 70, to further characterize the cellular immune response, we performed ELISPOT assays to quantify IFN-γ-producing splenocytes in immunized mice. Both sRBD and CRM-RBD immunization induced significant IFN-γ that showed dose-dependent enhancement. Notably, CRM-RBD immunization at 50 μg induced significantly higher spot-forming cell (SFC) counts than equivalent doses of sRBD, indicating enhanced Th1-type immunity (Figure 5D). Interestingly, the Th1 (IFN-γ) cellular immune response remained unaffected by RBD mutations regardless of immunization with either CRM-RBD or sRBD. We observed strong linear correlations between mutant and prototype RBD responses (Figure 5E). These findings demonstrate that CRM-RBD promotes a more balanced Th1/Th2 response compared to sRBD alone.

### CRM-RBD enhanced immune protection in mice post-challenge

To minimize experimental bias caused by RBD homology variations, we selected the Prototype and Delta variant (showing higher RBD homology with the prototype strain) for viral challenge to accurately evaluate the protective efficacy of the CRM-RBD vaccine. Mice immunized with either low-dose (25 μg) or high-dose (50 μg) CRM-RBD vaccine were challenged with the Prototype and Delta variant. Post-challenge monitoring included daily assessment of body weight and temperature fluctuations. On the final day of challenge (day 4 post-challenge for the prototype strain and day 5 for the Delta variant), we measured NAb titers, mortality and moribundity rates, and viral loads in the lungs.

In the Prototype and Delta variant challenge experiment, mice immunized with different doses of CRM-RBD exhibited significant protection. On day 3 post-challenge, the 50 μg CRM-RBD group showed the least body weight loss, followed by the 25 μg group, while the control group exhibited the most pronounced decline (Figures 3B,C). On the final day of challenge, the 50 μg group maintained stable body temperature, whereas the 25 μg group developed significant hypothermia (Figures 3D,E). Both immunization groups elicited significant NAb titers. Following prototype strain challenge, the 25 μg CRM-RBD group induced NAb titers of 57.72 GMTs, while the 50 μg dose group achieved 851.57 GMTs (Figure 3F). Against Delta variant challenge, the 25 μg and 50 μg CRM-RBD groups generated NAb titers of 51.45 GMTs and 384.00 GMTs, respectively (Figure 3G). All immunized mice survived, while the control groups for the Prototype strain and Delta strain showed mortality rates of 60% (6/10) and 32% (6/19), and moribundity rates of 20% (2/10) and 68% (13/19), respectively (Figures 3H,I). Viral load analysis demonstrated that the 50 μg CRM-RBD group significantly reduced viral replication in lungs (Figures 3J,K). In summary, CRM-RBD induced substantial NAbs, effectively mitigated infection-associated weight loss, thermoregulatory dysfunction, and mortality, while significantly suppressing viral replication in lungs.

### CRM-RBD significantly alleviates lung pathological changes caused by infection in mice

**Figure 6:**
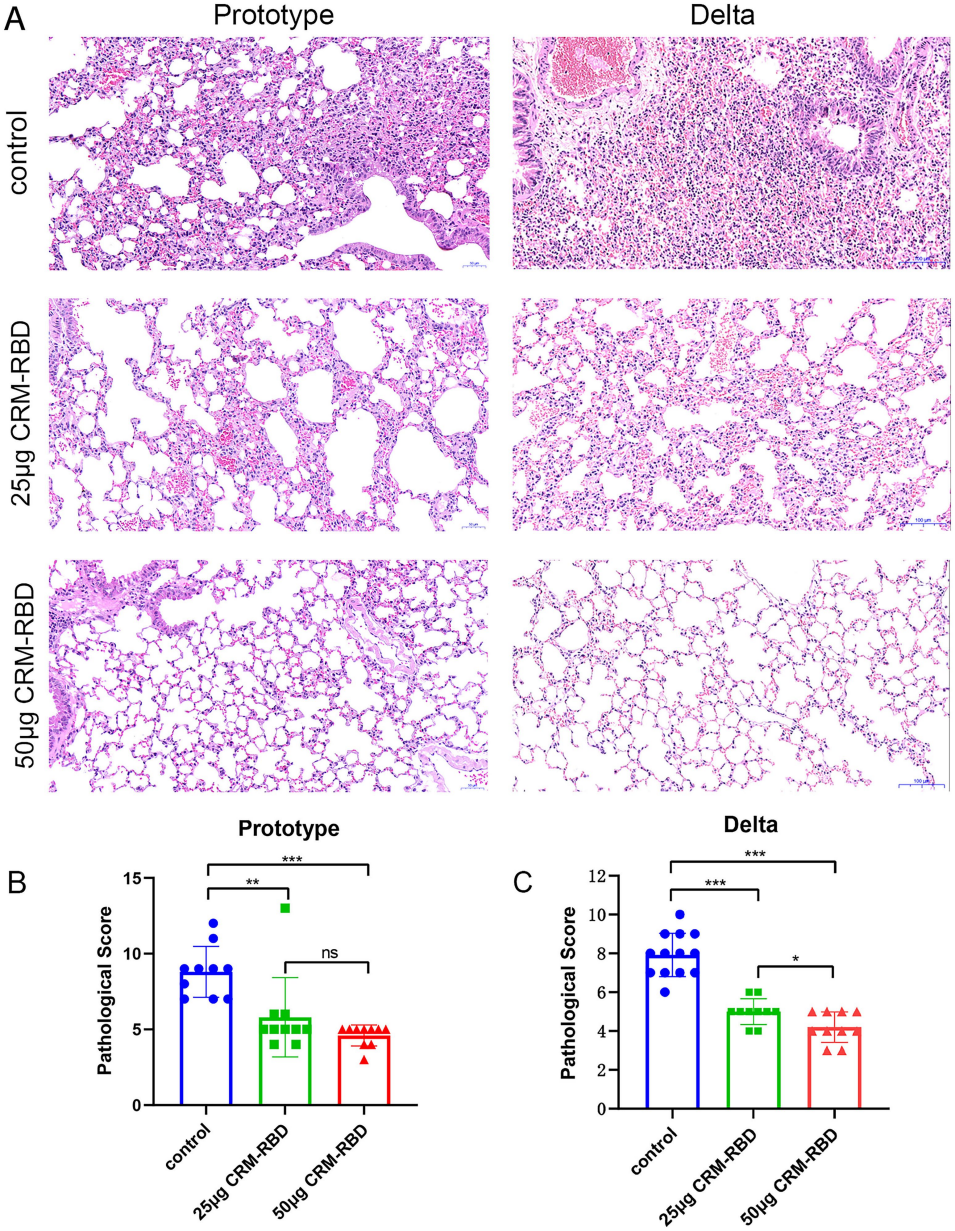
Histopathological observations in mice post-challenge. Following challenge, lung tissues from each group were dissected, fixed, sectioned, and subjected to HE staining for pathological diagnosis, with particular focus on histopathological changes. **(A)** Pathological manifestations of lung tissue damage in prototype strain and Delta variant groups. Prototype strain control group: Moderate to severe hemorrhage in most alveolar septa and some alveolar spaces; flocculent material adherent to bronchial lumens; focal rupture and fusion of alveolar septa forming large vesicles; localized alveolar space dilation; thrombus formation in vascular lumens; massive inflammatory cell infiltration. Prototype strain 25 μg CRM-RBD group: Mild to moderate hemorrhage in partial alveolar septa and some alveolar spaces; scattered blood cells adherent to bronchial lumens; mild edema and partial dissolution of alveolar septa; occasional intravascular thrombi; sparse inflammatory cell infiltration. Prototype strain 50 μg CRM-RBD group: Relatively intact alveolar structure with focal mild hemorrhage in septa; localized rupture and fusion of alveolar septa forming vesicles; minimal inflammatory cell infiltration. Delta variant control group: Hyperplasia of alveolar septal epithelial cells with localized thickening (predominantly mononuclear cells); moderate congestion and hemorrhage in alveolar septa; bronchial lumens obstructed by adherent histiocytes; thrombus formation; massive inflammatory cell infiltration (predominantly lymphocytes). Delta 25 μg CRM-RBD group: Mild hyperplasia of alveolar septal epithelial cells with localized thickening; mild congestion and hemorrhage in septa and alveolar spaces; sparse inflammatory cell infiltration (lymphocytes predominant). Delta 50 μg CRM-RBD group: Well-preserved alveolar structure; mild hyperplasia of septal epithelial cells; focal mild congestion in alveolar septa; minimal inflammatory cell infiltration (lymphocytes predominant). **(B,C)** Statistical results of pathological scoring for prototype strain. **(B)** and Delta variant **(C)** groups. Prototype strain: Control (*n* = 10), 25 μg CRM-RBD (*n* = 10), 50 μg CRM-RBD (*n* = 10). Delta variant: Control (*n* = 13), 25 μg CRM-RBD (*n* = 10), 50 μg CRM-RBD (*n* = 10). All experiments were performed in triplicate. Statistical significance: *p < 0.05, **p < 0.01, ***p < 0.001, ns (not significant).

Following challenge with different viral strains, lung tissues from each experimental group were collected for histopathological examination. In both the Prototype and Delta variant challenge groups, the control mice exhibited severe pathological damage, including extensive alveolar septal hemorrhage, bronchial lumen obstruction by cellular debris, intravascular thrombosis, and significant inflammatory cell infiltration. Mice immunized with 25 μg CRM-RBD showed moderate pathological lesions with mild inflammatory cell infiltration. Notably, the 50 μg CRM-RBD group displayed relatively preserved alveolar architecture, only mild localized tissue damage, and minimal inflammatory infiltration (Figure 6A). Statistical analysis of histopathological scores revealed that CRM-RBD immunization significantly reduced lung injury in both Prototype-and Delta-challenged groups (Figures 6B,C). These findings demonstrate that CRM-RBD effectively mitigates virus-induced pulmonary pathology in mice.

## Discussion

In this study, the CRM-RBD fusion protein was successfully expressed in *E. coli* and refolded *in vitro*. Compared to sRBD alone, CRM-RBD elicited more robust humoral immunity and Th1 cellular immune responses in mice, while simultaneously reducing immune evasion against SARS-CoV-2 Delta and Omicron variants. Importantly, CRM-RBD-immunized mice were protected against Prototype and Delta variant challenge, as evidenced by reduced viral loads in tissues and attenuated lung pathology. These findings suggest that CRM197 fusion represents an effective approach for developing broadly protective SARS-CoV-2 RBD vaccines. Furthermore, CRM197 functions as an intramolecular adjuvant capable of inducing broad-spectrum neutralizing antibodies, highlighting its potential as a candidate adjuvant protein for vaccines against highly pathogenic microorganisms.

The RBD of SARS-CoV-2, which contains the majority of neutralizing epitopes, represents an optimal target for subunit vaccine development (Dai et al., 2020). Previous studies have demonstrated successful production of recombinant RBD using various expression systems, including baculovirus, yeast, and *E. coli* (Chen et al., 2020; Shang et al., 2020; Yang et al., 2020; Liu L. et al., 2022). Based on considerations of production scalability and global accessibility, we selected the *E. coli* expression system for our SARS-CoV-2 subunit vaccine development. This choice is further supported by the proven clinical track record of *E. coli*-expressed subunit vaccines, as evidenced by the successful licensure of hepatitis E virus (HEV) and human papillomavirus (HPV) vaccines by China’s National Medical Products Administration (NMPA), which have demonstrated favorable immunogenicity, efficacy, and safety profiles in clinical trials (Hu et al., 2014; Zhang et al., 2015).

To date, emerging SARS-CoV-2 variants have not rendered current vaccines completely ineffective but have reduced their protective efficacy. This suggests the presence of conserved cross-reactive epitopes between the prototype strain and circulating variants. Enhancing the immunogenicity of these epitopes could therefore improve vaccine resilience against future mutations. In this study, we developed a CRM-RBD vaccine candidate to augment the immunogenicity of SARS-CoV-2 RBD-based vaccines. Both CRM-RBD and sRBD were successfully expressed in *E. coli*, predominantly as inclusion bodies, necessitating *in vitro* refolding to obtain properly folded proteins. As evidenced by Result 2 (Figure 4), the conformational parameters (λ_ex280nm_ Max and particle size) of both proteins exhibited alterations during the denaturation-renaturation process upon gradual removal of the denaturing agent. The proper folding of the proteins was subsequently verified through their binding affinity to hACE2. Notably, protein conformation prediction analyses revealed that the CRM-RBD fusion exhibited superior structural stability compared to the unfused counterpart. These findings collectively demonstrate that: The sRBD domain can be effectively refolded *in vitro*; The fusion with CRM197 not only preserves the renaturation efficiency but also confers enhanced stabilization to the tertiary structure of sRBD.

Consistent with the findings of Su et al. (2021), our study confirmed that *E. coli*-expressed sRBD, when adjuvanted with aluminum, elicits robust humoral and cellular immune responses. Notably, CRM-RBD demonstrated superior immunogenicity compared to sRBD alone, particularly in terms of humoral immunity. While both sRBD and CRM-RBD exhibited some degree of antibody-mediated immune escape against the Delta and Omicron variants, CRM-RBD showed significantly stronger resistance to immune evasion than sRBD alone (Figure 2D). We further assessed Th1 responses. CRM-RBD induced higher levels of IgG2a antibodies, a marker of Th1 polarization (Figure 5B). Additionally, ELISPOT analysis revealed that both CRM-RBD and sRBD triggered strong Th1-driven IFN-γ secretion, which remained unaffected by viral mutations (Figures 5D,E). These findings align with prior studies demonstrating that functional non-neutralizing antibodies and T-cell responses are largely preserved against SARS-CoV-2 variants post-vaccination (Alter et al., 2021; Liu J. et al., 2022).

Among all SARS-CoV-2 variants examined to date, Omicron exhibits the highest number of altered sites, with approximately 60 substitutions, insertions, and deletions, compared to only 22 mutations in the Delta variant (He et al., 2021). These mutations may contribute to increased transmissibility, higher reinfection risk, and reduced vaccine efficacy (Kumar et al., 2022). Notably, the Omicron RBD harbors 15 mutations, among which only L452R and T478K are commonly found in the RBD of the Delta variant (Chavda et al., 2022). Given this substantial divergence, we selected the Prototype and Delta strain for challenge experiments following CRM-RBD immunization to minimize potential confounding effects due to low RBD homology with the prototype strain. This approach allowed a more precise evaluation of CRM197’s adjuvant effect in enhancing vaccine protection against highly pathogenic pathogen. Among the numerous SARS-CoV-2 variants, Omicron has raised significant public health concerns due to its high transmissibility and antibody evasion properties, having rapidly evolved from BA.1 to BA.5 (Shrestha et al., 2022). Since early July 2022, BA.4/5 and their emerging descendant/recombinant lineages have been driving new pandemic waves (Wang et al., 2023). Moreover, other lineages, including the BA.2.86 subvariants JN.1, KP.2, and KP.3, have also played notable roles in global circulation (Wu et al., 2025). To further evaluate the vaccine potential of CRM-RBD, future studies should include challenge protection experiments against currently circulating strains. These investigations would enable comprehensive assessment of CRM-RBD vaccine’s efficacy and provide critical foundations for subsequent vaccine optimization.

## Data Availability

The original contributions presented in the study are included in the article/Supplementary material, further inquiries can be directed to the corresponding authors.
