# Supplementary material for: Enhancement of immunogenicity of SARS-CoV-2 spike protein expressed in Escherichia coli by fusion of the CRM197 functional domain
Source: Front Microbiol. 2025 Aug 12;16:1650239. doi: 10.3389/fmicb.2025.1650239 (PMC12378270; doi:10.3389/fmicb.2025.1650239)
Supplement: Supplementary file 1 [file Table_1.docx]

Table S1 sRBD and CRM197 sequences

| Sequence name | Sequence (5’- 3’) |
| --- | --- |
| sRBD | nitnlcpfgevfnatrfasvyawnrkrisncvadysvlynsasfstfkcygvsptklndlcftnvyadsfvirgdevrqiapgqtgkiadynyklpddftgcviawnsnnldskvggnynylyrlfrksnlkpferdisteiyqagstpcngvegfncyfplqsygfqptngvgyqpyrvvvlsfellhapatvcgpkkstnlvknkcvnfnfngltgtgvltesnkkflpfqqfgrdiadttdavrdpqtle |
| CRM197 | mgaddvvdssksfvmenfssyhgtkpgyvdsiqkgiqkpksgtqgnydddwkefystdnkydaagysvdnenplsgkaggvvkvtypgltkvlalkvdnaetikkelglslteplmeqvgteefikrfgdgasrvvlslpfaegsssveyinnweqakalsveleinfetrgkrgqdamyeymaqac |
