# Supplementary material for: Enhancement of immunogenicity of SARS-CoV-2 spike protein expressed in Escherichia coli by fusion of the CRM197 functional domain
Source: Front Microbiol. 2025 Aug 12;16:1650239. doi: 10.3389/fmicb.2025.1650239 (PMC12378270; doi:10.3389/fmicb.2025.1650239)
Supplement: Supplementary file 2 [file Table_2.docx]

Table S2 Primer sequences

| Primer name | Primer sequence (5’- 3’) |
| --- | --- |
| Forward primer | GACCCCAAAATCAGCGAAAT |
| Reverse primer | TCTGGTTACTGCCAGTTGAATCTG |
| Probe | FAM-ACCCCGCATTACGTTTGGTGGACC-BHQ1 |
