# Supplementary material for: Enhancement of immunogenicity of SARS-CoV-2 spike protein expressed in Escherichia coli by fusion of the CRM197 functional domain
Source: Front Microbiol. 2025 Aug 12;16:1650239. doi: 10.3389/fmicb.2025.1650239 (PMC12378270; doi:10.3389/fmicb.2025.1650239)
Supplement: Supplementary file 3 [file Table_3.docx]

Table S3 Scoring Criteria for Murine Pulmonary Pathological Alterations

| Score | Pathological Alterations |
| --- | --- |
| 0 | Intact alveolar architecture with no inflammatory infiltration |
| 1 | Mild inflammation; slight widening of interalveolar septa with sparse mononuclear cell infiltration (monocytes and lymphocytes) |
| 2 | Marked inflammation; thickened alveolar walls and increased mononuclear cell infiltration in the interstitium |
| 3-4 | Significant widening of interalveolar septa with increased density of inflammatory cell infiltration |
| 5 | Extensive exudation and septal widening; reduced alveolar spaces, evident interstitial hemorrhage, and increased cellular infiltration within alveolar lumens |
| >5 | Massive cellular infiltration obliterating alveolar spaces; confluent areas of septal fusion with hyaline membrane formation along alveolar walls |
